# Inclusion of children with disabilities in qualitative health research: A scoping review

**DOI:** 10.1371/journal.pone.0273784

**Published:** 2022-09-01

**Authors:** Janet Njelesani, Vongai Mlambo, Tsedenia Denekew, Jean Hunleth

**Affiliations:** 1 Department of Occupational Therapy, New York University, New York, NY, United States of America; 2 Stanford University School of Medicine, Stanford, CA, United States of America; 3 New York University Abu Dhabi, Abu Dhabi, UAE; 4 Division of Public Health Sciences. Washington University in St. Louis, St. Louis, MO, United States of America; De Montfort University, UNITED KINGDOM

## Abstract

**Background:**

Children with disabilities have the right to participate in health research so their priorities, needs, and experiences are included. Health research based primarily on adult report risks misrepresenting children with disabilities and their needs, and contributes to exclusion and a lack of diversity in the experiences being captured. Prioritizing the participation of children with disabilities enhances the relevance, meaningfulness, and impact of research.

**Methods:**

A scoping review was conducted to critically examine the participation of children with disabilities in qualitative health research. The electronic databases PubMed, PsychInfo, Embase, and Google Scholar were searched. Inclusion criteria included qualitative health studies conducted with children with disabilities, published between 2007 and 2020, and written in English. Articles were screened by two reviewers and the synthesis of data was performed using numeric and content analysis.

**Results:**

A total of 62 studies met inclusion criteria. Rationales for including children with disabilities included child-focused, medical model of disability, and disability rights rationales. Participation of children with disabilities in qualitative health research was limited, with the majority of studies conducting research *on* rather than in partnership *with* or *by* children. Findings emphasize that children with disabilities are not participating in the design and implementation of health research.

**Conclusion:**

Further effort should be made by health researchers to incorporate children with a broad range of impairments drawing on theory and methodology from disability and childhood studies and collaborating with people who have expertise in these areas. Furthermore, an array of multi-method inclusive, accessible, adaptable, and non-ableist methods should be available to enable different ways of expression.

## Introduction

Childhood studies, an interdisciplinary field comprised of researchers from the social and humanistic sciences, have established that young people are social actors and that children’s participation in research provides a necessary form of research evidence [1,2]. Even though children with disabilities’ participation in research has increased in recent years, studies of their health tend to remain adult-centric, in part because of “the entrenched protectionist and paternalistic perspectives that have historically pervaded disciplines such as medicine” [3]. Not widely capturing the perspectives of children with disabilities contributes to exclusion and a lack of diversity in the health experiences being captured [4].

Understanding children with disabilities’ experiences from their perspective is particularly important when addressing their health concerns, and when designing health programming to fit their needs. Health research on children with disabilities based solely on adult report risks misrepresenting children and their needs for multiple reasons [5]. Adults, including caregivers, do not always know what their child knows, understands, or experiences, and they might hold different understandings of children’s subjective well-being [6]. Most critically, adults, even caregivers, do not have access to all of children’s experiences both because they do not have access to children’s embodied experiences and children engage in many activities apart from their caregivers [7]. Children may not report these experiences and, in some cases, may actively conceal their knowledge, perspectives, and experiences from caregivers for a variety of reasons, including to protect them [8].

In this review, we focus on children with disabilities for several reasons. First, the health and mortality of children with disabilities are gaining visibility across health fields [9–11]. Second, childhood and disability studies scholars have developed sophisticated methodological and theoretical insights on children with disabilities’ research participation and demonstrated that children with disabilities can participate in research and offer unique insights on their worlds [4,12].

The participation of children with disabilities enhances the relevance and positive impact of research [13,14]. Such engagement, especially through qualitative approaches, is seen as crucial for understanding context and increasing the value of research to benefit both researchers and communities. However, how health researchers are including children with disabilities in research is unknown. Existing reviews have not focused on the participation of children with disabilities in health research [9,15,16]. Without explicit attention, research methods can be ableist and exclusionary and fail to illuminate issues relevant to children with disabilities [17]. Having such an understanding would allow researchers to identify and avoid practices that exclude and marginalize children with disabilities. Furthermore, it would enable health researchers to recognize how to best include children with disabilities to actively participate in research that concerns their everyday lives.

### Conceptual framework: Research on, with, or by children

We consider participation as any time children were included as research participants, and we use an understanding of participation from childhood studies to differentiate how children with disabilities participated in such research. Specifically, we draw on a conceptual framework that categorize approaches to children’s participation in research into three conceptual categories: *on*, *with*, or *by* [18]. The use of the prepositions *on*, *with*, and *by* denotes dominant traditions in childhood research that hold underlying assumptions about children’s roles in the research process. Historically, research *on* children has been the dominant mode, where children are the objects and not the subjects of research. Research *on* children typically does not include children as active participants, but rather collects information about children through other means, such as through interviewing adults. At times, children are invited to participate in research, but their participation may be tokenized or manipulated [19], and we define this too as research *on* children in this study.

Counter-acting such trends, childhood studies scholars have advanced the notion of research *with* and also *by* children, see for example Christensen and James [1]. This marked a paradigm shift to considering children as active and thinking contributors within research studies. Research with children encompasses a range of ways of engaging children using verbal, visual, and participatory methods. Children have been included in such work, from standard interviews or focus group formats to the integration of arts-based and activity-based methods and the adaptation of methods to fit with children’s preferences. There is no one best method, but rather a commitment to tailoring methods to facilitate a better understanding of children’s experiences from their perspectives. Research *by* children involves inviting children to participate in shaping the research agenda as researchers.

The empirical and theoretical underpinnings promoting this shift from research *on* children to research *with* and *by* children include childhood studies’ focus on childhood experiences as diverse cross-culturally that challenge previous ideas about “normal” stages of development, and children as agents and children’s interdependencies that show that children, too, influence adults and shape their immediate and wider social worlds [20]. Children’s participation was also prompted by Article 12 of the United Nations Convention on the Rights of the Child (CRC), that every child should have the right to freely express their views.

### Review aim

As a result of the limited knowledge on the participation of children with disabilities in health research, this scoping review aimed to generate new insights that can be used to influence research and thereby increase opportunities for children with disabilities to participate meaningfully in health research. This is vital to advancing health research by ensuring it is representative, comprehensive, and relevant to children with disabilities’ lives.

## Methods

A scoping review was completed to systematically review the participation of children with disabilities in qualitative health research. The Preferred Reporting Items for Systematic Reviews and Meta-Analyses Extension for Scoping Reviews (PRISMA-ScR) [21], was used in order to improve the methodological and reporting quality of this review (see S1 File). The protocol for this study was not registered. We did not critically appraise the studies as scoping reviews do not focus on quality assessment [22].

### Data sources and searches

Working with a health sciences librarian we developed our search strategy. Using combinations of the following keywords and related MeSH terms: children; disability; participation; health; and qualitative research, we searched the electronic databases PubMed, PsychInfo, and Embase. Sample search strategies can be found in S2 File. We also conducted a Google Scholar search to identify studies not included within the databases for articles published from January 2007 to December 2020. Only the first 100 citations from Google Scholar were included because Google Scholar retrieves citations that are ordered by their relevance to the search topic. Given that 2007 is the year after the coming into action of the United Nations Convention on the Rights of Persons with Disabilities (UN CRPD), we only searched for articles published since 2007. Inclusion criteria included peer-reviewed qualitative or mixed-methods published health studies that included children with disabilities [i.e., children who have long-term physical, mental, intellectual, or sensory impairments which in interaction with various barriers may hinder their full and effective participation in society on an equal basis with others] [23] as study participants, and were written in English. Articles that exclusively used quantitative methods, focused on health conditions that were not described as a disability in the study, not related specifically to health (e.g., education outcomes with no reference to health), review articles, grey literature, and opinion pieces were excluded. Articles were not excluded if in addition to children with disabilities they also included parents, adults, and non-disabled children as study participants.

### Study selection

Studies were selected via a two-step process using EndNote X8. First, two reviewers screened all of the titles and abstracts for inclusion and exclusion criteria. Then, full-texts were read by two reviewers independently to confirm if they fulfilled the inclusion and exclusion criteria. Disagreements at each stage were resolved by a third person. The same set of inclusion and exclusion criteria was used for both levels of screening.

### Data extraction

Data from each of the included articles were extracted into a spreadsheet. Data were extracted according to the review aim and included; author’s name, publication year, study location, first author’s department affiliation, study design, study aim, study methods, participant age, participant diagnosis, rationale for including children with disabilities, barriers to including children with disabilities, and ways that children were involved in each study. Four reviewers extracted data and 50 percent of the articles were double extracted with controversy and ambiguities resolved during team meetings.

### Data analysis

Data analysis included numeric and content analysis [24]. Numeric analysis focused on quantitatively summarizing study characteristics, types of methods used, and characteristics of children who participated in the research. Descriptive content analysis was used to synthesize non-numerical data. The ways that children were involved in each study as described in the methods sections was categorized according to Mason and Watson’s criteria [18]. To ensure rigor, both analyses were completed by two researchers who wrote analytical notes throughout the process to document emerging patterns. The final categories were confirmed by a third member of the research team.

## Results

### Study selection results

**Fig 1:**
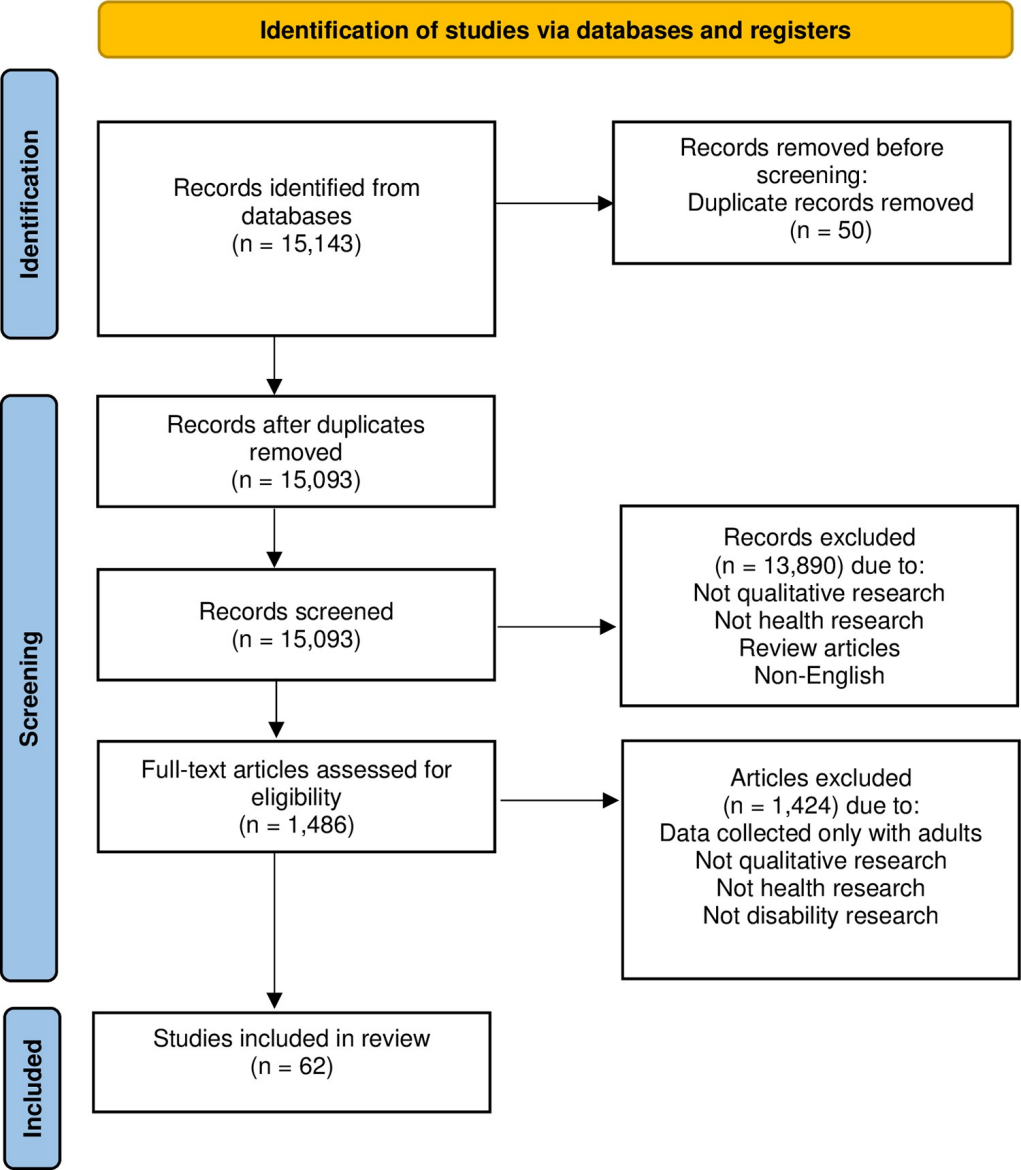
PRISMA flow diagram.

The search strategy yielded a total of 15,093 articles after the removal of duplicates. Manual screening of titles and abstracts to exclude articles that were not qualitative, mixed-method research reporting qualitative evidence, or focused on health left 1,486 articles for full-text review. All articles that focused solely on non-disabled children or adults’ (e.g., parent, guardian, health provider) experiences were excluded and as a result, 62 studies were included in the final analysis [25–87]. This process is documented in Fig 1 as per the PRISMA-ScR guidelines. A summary of study characteristics for the studies included in this review is presented in Table 1 and expanded descriptions of the studies are provided in S1 Table.

**Table 1:** Study characteristics.

Study Characteristics	No. of Studies	% of Total
Year of Publication		
2007–2013	38	61.29
2014–2020	24	38.71
Study Locations		
North America	31	50.00
Europe [including UK]	17	27.41
Asia	7	10.45
Australia and New Zealand	4	5.97
Central and South America	2	2.99
Africa	1	1.49
Age of Youngest Child		
2–5	10	16.12
6–9	28	45.16
10–13	11	17.74
14–17	10	16.12
not stated	3	4.84
Conditions/Impairments*		
Neuromusculoskeletal and Movement Related	27	43.55
Mental Functions	18	29.03
Functions of the Cardiovascular, Hematological, Immunological and Respiratory Systems	4	6.45
Digestive, Metabolic and Endocrine Systems	3	4.84
Sensory Functions and Pain	2	3.23
Voice and Speech Functions	1	1.61
Genito-urinary and Reproductive Functions	0	0.00
Functions of the Skin and Related Structures	1	1.61
Not Specified	8	12.90
Form of Inclusion		
Research *on* children	47	75.81
Research *with* children	10	16.13
Research *by* children	5	12.40

*Specific conditions classified using the International Classification of Functioning, Disability and Health [ICF] and ICD-10 codes.

### Characteristics of included studies

All 62 studies were published in peer-reviewed journals between 2007 and 2020. Studies took place in a total of 18 countries. However, the top two study locations in descending order were Canada and the United States of America, which represented 50% (n = 31) of the articles reviewed. The academic departments of the first author were diverse. From the 57 studies where the first author’s department affiliation was explicitly reported, 32 different fields were represented, with the highest number of studies from departments of nursing (n = 6), psychology (n = 4), pediatrics (n = 4), and physical therapy (n = 4).

Our review population of interest was children with disabilities. Twenty-five studies exclusively involved children with disabilities as research participants. All remaining articles involved children with disabilities and also collected information from nondisabled children, caregivers, parents, siblings, teachers, or medical professionals. While all studies included both girls and boys with disabilities, the ages and impairments of these children varied. Ages of children ranged from 2 to 18 years old and 34 different diagnoses were represented, including cerebral palsy (n = 13), autism (n = 5), juvenile idiopathic arthritis (n = 4), attention deficit hyperactivity disorder (n = 4), mental health disorder (n = 4), traumatic brain injury (n = 3), and complex chronic conditions (n = 2). Additionally, studies took place in diverse settings in homes, schools, youth centers, hospitals, and clinics. The majority of studies used interviews (n = 56) to generate data, with focus group discussions (n = 17) also frequently used. Eighteen studies included arts-based approaches (i.e., visual arts, drama, music) as part of the interview or focus group process. For example, Pauschek et al [25] used children’s drawings to gain insight into their experiences of epilepsy with pictures being “an additional means of communication and self-expression”. Similarly, a study by Yeung et al [26] used a pediatric body map during interviews to explore how children described their neuropathic pain.

The objectives of the studies were diverse and generally included exploring the subjective experiences of children living with their impairments, accessing mental health or hospital-related services, participating in physical activities, and taking part in intervention programs. Overall, articles were focused on research about the children’s impairment rather than research that explored the overall health and wellness of children with disabilities. For example, Merrick et al. [27] interviewed children with speech, language, and communication needs with a targeted focus of understanding how the children perceived communication challenges and assistance, to the exclusion of other aspects of their health that may be salient and inter-related. Few articles focused on the overall health and wellness considerations of children with disabilities. Suarez-Balcazar et al [28] explored barriers faced by youth with disabilities in pursuing a healthy lifestyle, such as access and affordability of healthy foods, opportunities to engage in physical activity, and availability of transportation to playgrounds. This study also went beyond an impairment-specific focus by including a range of children with different impairments.

### Rationales for including children with disabilities

Researchers’ underlying assumptions shaped how children participated and were treated in research. We examined the articles for researchers’ reported rationales for including children with disabilities in their health research. Five articles did not provide any rationale. Authors of the remaining articles offered explicit statements, and through our analysis, we identified three mutually inclusive rationales: 1) child-focused rationale, 2) medical model of disability rationale, and 3) disability rights rationale. Some articles (n = 17) alluded to more than one of these rationales.

#### Child-focused rationale

The majority of studies (n = 36) stated understanding the lived experiences of children with disabilities from their perspective as their primary reason for inclusion. These articles acknowledged children as independent social agents whose health experience cannot adequately be captured by adult proxies, even if these individuals are intimately involved in their care. Further, these studies contended that children are knowledgeable and understand more concepts about their health and illness than previously assumed [29]. For example, Gibson et al [30] posit that “children’s perspectives may not be represented by their parents so requires parallel exploration using child-centered methods”. Similarly, Speraw et al [31] not only characterized children as independent social stakeholders but also experts who provide crucial contextualization of their lives. As such, their study aimed to “see beyond the healthcare providers and outside stakeholder’s opinions about the objective quality criterion of a functional life and take the young respondent’s perspective on what life is and ought to be like” [31]. Although these studies highlighted seeking children’s perspectives as critical and despite orienting their work in childhood studies, theoretical support was lacking as few articles referenced theories or frameworks to support the inclusion of children in their research (e.g., theory on children’s agency).

#### Medical model of disability rationale

Twenty-three studies were oriented to a medical model of disability rationale (i.e., a model of disability that sees disability as the result of a physical condition, is focused at the individual level, identifies people by their diagnosis, and believes disability can be cured by health specialists). The specific rationales under this sub-theme varied by type of impairment. For example, a recurring rationale for research involving children with cerebral palsy was to gain insight into barriers and facilitators to engaging in physical activity [32–34]. Rationales for including children with arthritis focused on using their perspectives to improve hospital-based treatment outcomes, illness management, and transition care plans [35–37]. Most studies that used the medical model rationale were condition-specific and more narrowly oriented towards addressing challenges in the medical management of children’s impairments.

#### Disability rights rationale

Twenty studies explicitly highlighted that inclusion of children with disabilities in health research will address the chronic under-representation and disparities disproportionately faced by this demographic. These articles either directly stated or alluded to the right of children with disabilities to participate and access the benefits from being included in the research. The benefits stated for children participating in the research were a sense of inclusion, feelings of companionship with children who share similar disabilities in studies with group interactions, and empowerment to be active, vocal research participants.

Several authors described how invisibility in literature is a form of marginalization and sought to use their study as a way to remediate these gaps in research. On the topic of social inclusion, Lindsay et al [38] noted that “[social inclusion] is under-researched for children and youth with disabilities even though they are bullied and excluded at disproportionately high rates”, while a study by Tveten et al [39] on goal-directed physical rehabilitation suggested that increased understanding garnered from their research “may contribute to reduced disparities”. Therefore, these articles show an awareness of how inclusion can address existing, exclusionary social structures that are reflected in research practices. Additionally, five of these articles also explored how dominant social norms and values impacted the children’s experiences. These studies contextualized children in their social environment and explored salient interactions that shape children’s perspectives about themselves and notions of disability.

### Focus on inclusion in research

We assessed the level and manner of child inclusion in the research using Mason and Watson’s [18] three categories of inclusion: 1) research on children; 2) research with children; 3) research by children.

#### Research on children

This level of inclusion involved minimal engagement with child participants. The majority of articles (n = 47) fell in this category. Even though studies aimed to carry out research with children, the articles positioned children in passive ways. They did so primarily through relying on adult interviews with parents for data generation about their children. For example, parents were asked questions regarding their perceptions of outpatient care experiences for their children [40]. In this study, children were interviewed, but their interview data was considered informal, thereby deprioritizing their input. As such, adult investigators retained discursive authority over the quotes acquired from children. Articles in this category mostly used interviews and focus groups to collect data and did not offer different ways for children to tell their experiences such as through visual or activity focused methods.

#### Research with children

Research *with* children treated participants as knowledgeable social agents. When research was done “with” children, they actively participated and shared their views, concerns, and ideas not only on the research topic but also on the research process itself. The manifestation of this research practice varied but usually involved minimizing and critiquing adult-centric perspectives by soliciting child input on the efficacy of methods used or the accuracy of data collected. Ten articles fit into this category. Notably, engagement with children in the research process primarily took the form of member-checking, where the researcher presented preliminary interview guides, methods, or findings to participants or a children’s advisory board to assess its salience and accuracy (n = 7). For example, Willis et al [41] established a steering group including adolescents with disabilities to provide feedback on the interview guide and to review transcripts generated. Similarly, Giarelli et al [42] asked child participants to assess the credibility of the conceptual model they derived.

#### Research by children

Research *by* children encouraged children to participate in conducting the research itself. Children were considered research partners and helped lead the study. Few studies (n = 5) met the threshold of this category and are presented in Table 2. In this research, children were engaged in diverse research tasks such as identifying the research aim, method development, data interpretation, and dissemination. As a result, these studies tended to incorporate more participatory and multi-modal data collection methods to increase engagement including the integration of arts-based methods. In Coad and Coad’s study [43] children on the advisory group were trained in key research processes to further enable their contribution to the study. In Gibson et al. [44] youth advisors helped to revise protocols so that they would include more inclusive data generation techniques such as emailing questions to participants beforehand so they could answer at their own pace.

**Table 2:** Articles demonstrating research *by* children.

Citation	Study Aim	Description of Child Participation	Methods
Coad & Coad [43]	To explore young people’s views and preferences of thematic design and color in the hospital environment.	A youth advisory group was formed and they were trained on research processes. The advisory group informed several aspects of the research including study design, piloting, questionnaire development, data collection, and coding of interviews.	Interviews, focus groups, arts-based
Ngo et al. [45]	To examine the lived experiences of children with disabilities in an Agent Orange affected region in Vietnam.	An advisory board that included youth union representatives was created. Youth participated in identifying topics for investigation, developing sample selection, and recruiting and training local investigators.	Interviews, focus groups
Moyson & Roeyers [65]	To investigate how siblings of children with intellectual disability define their quality of life as a sibling.	Child participants determined whether findings matched what is important to them and had an opportunity to revise including deleting anything they did not want included in the research.	Interviews, drawing, play
Gibson et al. [44]	To develop a better understanding of the interacting socio- material and personal forces that shape activity participation.	Child participants who participated in both phases of the two-part study served as an advisor during the research. Interim reports were shared with stakeholders to inform subsequent interpretations.	Photo elicitations, observation, interviews
Montreuil et al. [64]	To examine the experiences of children related to conflict and crisis management and the use of restraint and seclusion in a mental health setting.	An advisory committee of children receiving care were consulted to make key decisions about study questions, data collection, analysis, and dissemination.	Participant observation, interviews

### Barriers to participation in research

A total of 27 articles indicated barriers to including children with disabilities in their studies as research participants (i.e., research done on children) or as research partners (i.e., research done with or by children). Barriers most often cited (n = 23) were at the level of the child, such as a child’s age or a child with a learning disability or communication impairment assumed to be unable to respond to questions. This was seen in articles that reported how participants had difficulty articulating their ideas in interviews and appeared to be related to young age (less than 7 years) and reported learning difficulties [32]. Ngo et al [45] included participants with physical and sensory disabilities and excluded children with intellectual and psychiatric disabilities, mentioning “limited project timeframes and lack of methodological expertise in research with people with intellectual disabilities” as barriers to inclusion. Another study excluded young children with disabilities, noting “The interviews with two young children (i.e., 4 and 7 years of age) were excluded from the analysis, because the children were unable to clearly articulate their experiences” [87]. However, the data collected from their parents and healthcare providers were included in the analysis. These cited barriers overall contributed to a lack of representation from participants of younger ages and across impairment types.

## Discussion

This review provides a novel assessment of the current state of inclusion and the actions that could be employed to further advance the participation of children with disabilities in health research. Currently, most of the systematic reviews of this nature focused on persons with disabilities are limited to the participation of adults, which are also finite in number [16,88]. Even beyond the scope of health research and children with disabilities, Bradbury-Jones et al. found only 13 articles that used a participatory approach with vulnerable youth of which children with disabilities were considered a subset [89] and Feldman et al [15] indicated that from published child development research articles, 89.9% excluded children with disabilities as participants. Although their inclusion criteria differed from ours, these studies indicate a consistent trend. Whether the scope is narrow or broad, children with disabilities are participating in research at abysmally low rates. Our review showed that there is a dearth of inclusion of children with disabilities in qualitative health research, with only 5 articles demonstrating research by children with disabilities, and 10 studies demonstrating research with children with disabilities. Health researchers are therefore inadvertently contributing to societal exclusion of children with disabilities by failing to include their experiences to inform research and decision-making processes that directly affect their lives. Not only does the exclusion of children with disabilities from research go against a rights-based perspective but also from a scientific ethics perspective researchers have a moral imperative to include children with disabilities as the findings are the foundation for evidence-based healthcare [90].

Health research has traditionally taken a paternalistic approach, with investigators as the gatekeepers of the knowledge production process. This effect is amplified when working with children, and even more so with children with disabilities, who face expectations about the degree to which having a disability and being a child affects their full participation [91]. Additionally, caregiver responses to interviews served to overshadow, rather than complement reports from children themselves [3,49,51]. Consequently, health research *with* and *by* children with disabilities helps to subvert these traditional hierarchies and positions children to be shaping investigations related to their well-being. Failure to ensure this level of inclusion results in losses for children with disabilities, health researchers, and practitioners who make use of this literature.

The rationale for including children with disabilities as participants were clearly articulated in almost all of the articles reviewed. Researchers overwhelmingly valued understanding child perspectives autonomously from other individuals in their lives. However, our review demonstrates that this inclusion is only the beginning of a truly equitable representation of children with disabilities in health research. The studies did not consistently reference child or disability rights literature despite their notable salience to the work, indicating a poor integration of disability studies literature to inform the research. This lack of integration may have the unintended consequence of perpetuating the gap between disability studies and health research when this intersection could prove highly beneficial to children with disabilities [92].

The rationales provided by articles that fit into the medical model of disability illustrate the potential effects of siloed research. In this category, the motivation for including children was related to their specific condition, rather than a commitment to incorporate children with disabilities in general health research. As a result, when children are participants in research about nutrition, mental health, or sexual and reproductive health, those with disabilities were usually not recruited unless the health topic was linked to their impairment. Condition-specific research often fails to acknowledge children with disabilities as holistic individuals with diverse health needs and has the potential to harm “when their research contributions are reduced to align with researchers’ focus on singular aspects of that child’s life experiences (e.g., living in poverty or experiencing a particular illness)” [93].

To better understand the participation of children with disabilities in health research, our study looked into not only how they were included, but also notable exclusion criteria as children with disabilities are too often excluded from research because they are perceived to be vulnerable or incompetent [94]. Findings from this review reveal that young children and children with learning disabilities or communication impairments were often not eligible to participate due to cited logistical challenges and lack of use of appropriate methods to communicate with respondents. This is consistent with previous research which shows that individuals, including children, with complex developmental and physical disabilities, have historically been excluded from direct action research [95]. This discrimination from research based on disability has a cascading effect, furthering manifesting itself in marginalization from resources and decision-making that results from the research evidence.

### Designing for greater participation in health research

Findings of the review provide several insights for health researchers. The first underscores that for research to be done *with* and *by* children, they need to play an active part throughout the life of the research. Studies that involve children at the onset of the research project to completion reap significant rewards as it enables children’s agendas and amendments to be central, rather than peripheral to the research agenda. However, for this to occur meaningfully, research teams need to commit to training children in an age-appropriate way, similar to how research assistants are equipped with the skills that they need to engage deeply with the project. The reflective guide developed by Hunleth et al. is one tool that may assist researchers who wish to deepen the meaningfulness of children’s participation in health research [96]. Although this preparation may require more time and resources, it will ultimately build children’s capacity that can be engaged in future research projects and ensuring that research aims are aligned with needs prioritized by children with disabilities.

Participation of children with disabilities in research requires planning and adaptation to align the research process and methods with participant’s abilities, preferences, and communication styles [97]. Thus, to conduct health research *with* and *by* children with disabilities, the research may need to be adapted as pre-existing prevailing norms and standards of how research is conducted may not be appropriate to use. This review, therefore, suggests the need for health researchers to incorporate more accessible, inclusive, and non-ableist research methods. One strategy is enhancing the accessibility of the research tools. This can partly be achieved by integrating technology, which could use assistive and augmentative communication devices children may already be familiar with. One tool that has already been created is Audio Computer Assisted Self-Interview (A-CASI), which employs user-controlled text, audio, and video content to collect data [95]. A-CASI’s adaptability enhances the agency of participants because participants can change the interview experience into a form that is most accessible to them.

The majority of researchers used interviews to collect data with fewer using other forms of expression, including visual and arts-based methods. The reliance solely on interviews has several critical limitations. It is recognized to be ableist, as using only verbal forms of communication privileges children with speech and excludes children who prefer to communicate in other ways [2]. What was lacking in the majority of research were the acknowledgments of the diversity of children’s experiences, how those experiences connect to children’s social and political contexts, and how the methods co-constructed knowledge between adults and children [98]. We recognize that research is complex and each method has its limitations, and therefore advocate for children to be actively involved in the decisions and co-construction of an array of methods that recognize the diversity of children’s abilities, preferences, and communication styles. In particular, from the findings of this review, we recommend for health researchers to go beyond relying on verbal interviews and consider incorporating more arts-based and visual methods and interactive techniques (e.g., music, puppets, drawings, photographs). These diverse methods may enable children to feel more comfortable to express their views and explore new meanings [99]. One example from the studies reviewed including Coad and Coad’s [43] work who went beyond verbal interviewing by using photographs and coded color leaflets to gain insight into children’s design preferences for their hospital environment. Their study also included young people on the project’s advisory board to inform research design, piloting, and analysis.

Further complementing the idea of using different modes of expression, is how research methods can be adapted to be inclusive of participants’ varying abilities. While there is a body of literature on enhancing participation of children in research, few have focused on children with disabilities. Recognizing this gap, a methodological approach that includes innovative techniques, strategies, and methods for engaging children with disabilities in qualitative research was developed [12]. The approach emphasizes assembling a range of customizable interview methods, partnering with parents, and consideration of the power differential inherent in research. Key is the acknowledgment that generated data is co-constructed and multiple methods that build on the strengths of the child may be incorporated.

Employing literature from childhood and disability studies will add to both the intellectual rigor and efficacy of children with disabilities’ engagement in research. While there is a deficiency in participatory research studies with children with disabilities, childhood, and disability studies fields could offer practical guidance on how to ethically work with children with disabilities as well as highlight critical theory that should be incorporated to contextualize the findings. This will significantly improve the synergy between health research and childhood and child disability studies to the benefit of all fields and the children this research is designed to support.

For the above actions to occur, and research aimed at examining pediatric health issues with children with disabilities to increase, persons with disabilities need to be prioritized in the agendas of political, academic, and scientific bodies. With this prioritization, greater resources will be allocated so researchers may have the means and time to collaborate and develop tools to conduct participatory research. In addition to funding sources, other mechanisms such as support from institutional research ethics review boards including board members with disabilities and child research expertise are needed.

### Limitations

In conducting this scoping review, we relied on the information contained in the articles. However, descriptions of methods related to children’s participation in the research were not always clear. Some common reporting approaches that made it difficult to assess children’s inclusion included: a lack of information about the methods used; the exclusive use of adult quotes; and the conflation of child and adult responses. Such practices in a few articles not only made it difficult to understand how children with disabilities participated in research but also diminished their contributions to the research and the benefits derived from including them. This highlights the importance of health researchers including explicit details about their methods of inclusion as this will enable greater knowledge sharing and consolidation of best practices.

Due to the limited number of studies that are classified as research *by* children and the scope of our review, we did not conduct an extensive analysis of the benefits derived from including children with disabilities. It would be valuable to comprehensively assess the impact of inclusion on researchers and children on key metrics important to both stakeholders, similar to how benefits were assessed for the inclusion of individuals with intellectual disabilities as co-researchers [88].

## Conclusion

The findings from our scoping review illuminate how health researchers’ underlying assumptions shape how children with disabilities participate and are treated in research. Overall, the findings suggest that children with disabilities are not sufficiently participating in the design and implementation of qualitative health research. They are, for the most part, included as passive sources of data whose perspectives are not being employed to inform the process of research. Key to the exclusion of children with disabilities from health research were researchers’ orientation to the medical model, assumptions that child factors (i.e., type of impairment) hinder participation, inadequate research accommodations, and lack of adaptation of research methods. To address existing, exclusionary structures that are reflected in research practices and increase participation, multi-method inclusive, accessible, adaptable, and non-ableist research tools should be used. Further effort should be made by health researchers to incorporate children with a broad range of impairments drawing on theory and methodology from disability and childhood studies.

## Supporting information

S1 Table Included publications. (XLSX)

S1 File Preferred reporting items for systematic reviews and meta-analyses extension for Scoping Reviews (PRISMA-ScR) checklist. (DOCX)

S2 File Search strategy. (DOCX)
