# Supplementary material for: Inclusion of children with disabilities in qualitative health research: A scoping review
Source: PLoS One. 2022 Sep 1;17(9):e0273784. doi: 10.1371/journal.pone.0273784 (PMC9436059; doi:10.1371/journal.pone.0273784)
Supplement: S2 File — (DOCX) [file pone.0273784.s003.docx]

**S2 File. Search strategy examples**

Databases: Embase, PubMed

Search date: Until 31 December 2020

**Embase**

(qualitative OR ethnography OR narrative OR interview) AND health AND (child* OR youth OR pediatric OR adoles* OR boy* OR girl* OR minor* OR juvenil* OR teen OR pubescen*) AND (participat* OR inclusion OR ‘action research’ OR ‘collaborative inquiry’ OR ‘community driven’) AND (disab* OR impair* OR disorder OR delay OR ‘mental disorder’ OR blind OR deaf OR Autis*)

**PubMed**

((“qualitative research”[MeSH] OR (“qualitative”[All Fields] OR “ethnography” [All Fields] OR “narrative” [All Fields] OR “interview” [All Fields])) AND ((“child health”[ MeSH] OR “adolescent health”[MeSH] OR “health”[All Fields]) AND ((“child”[MeSH] OR “adolescent” [MeSH] OR “pediatric” [MeSH] OR (“child”[All Fields])) AND ((“community based participatory research"[MeSH] OR (“action research”[All Fields])) AND ((“disabled children”[MeSH] OR (“disability”[All Fields]))
